# Supplementary material for: MiR-574-5p activates human TLR8 to promote autoimmune signaling and lupus
Source: Cell Commun Signal. 2024 Apr 8;22:220. doi: 10.1186/s12964-024-01601-1 (PMC11000404; doi:10.1186/s12964-024-01601-1)

Full unedited gels for Figure 2***a***


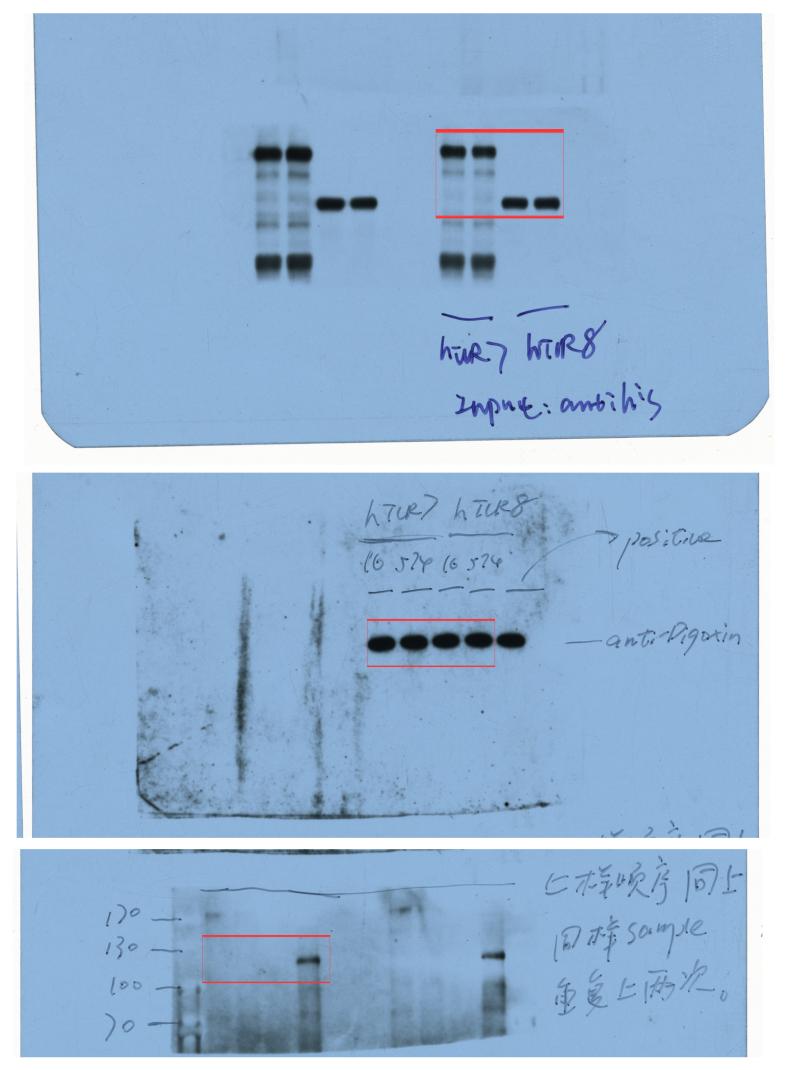


Full unedited gels for Figure 2***b***


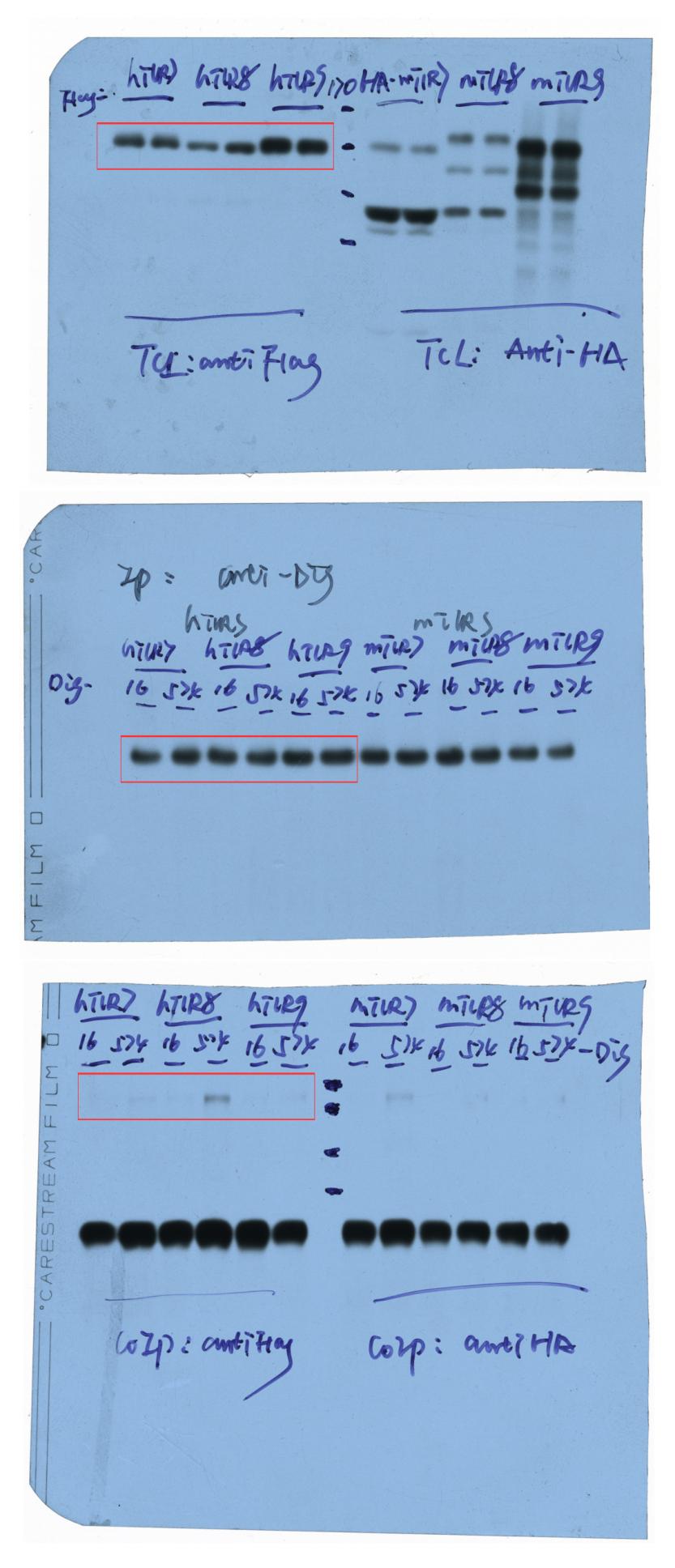


Full unedited gels for Figure 2***c***


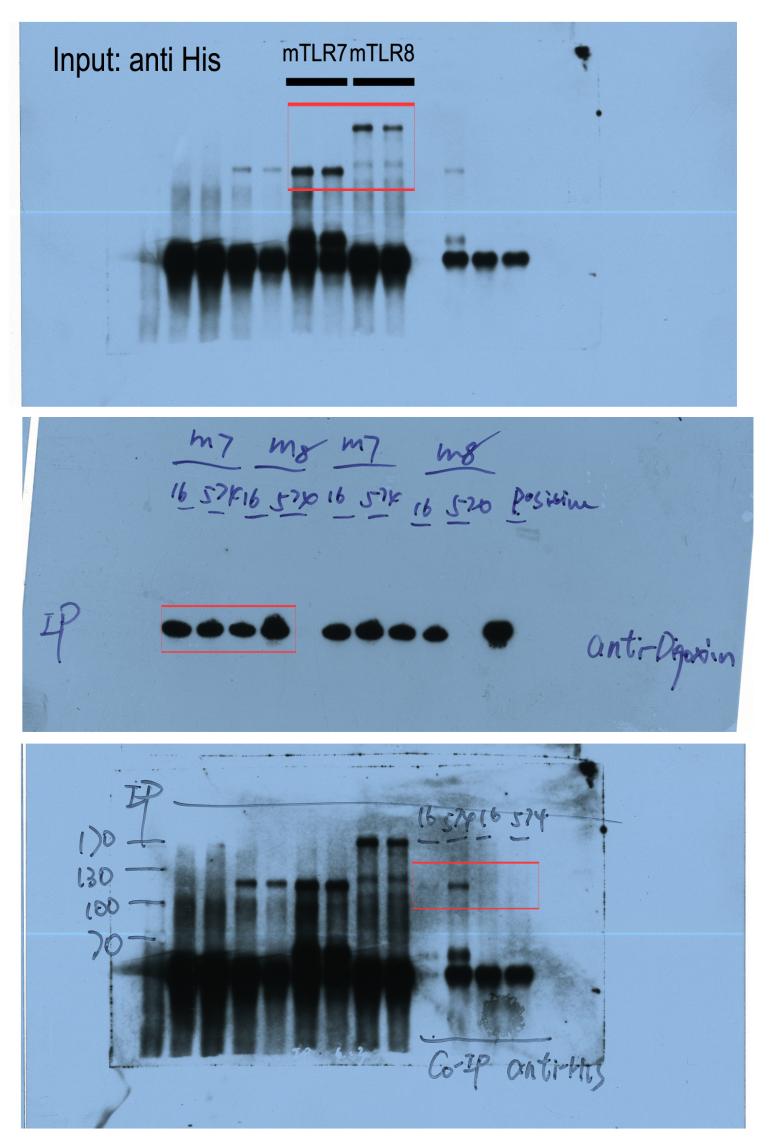


Full unedited gels for Figure 2***d***


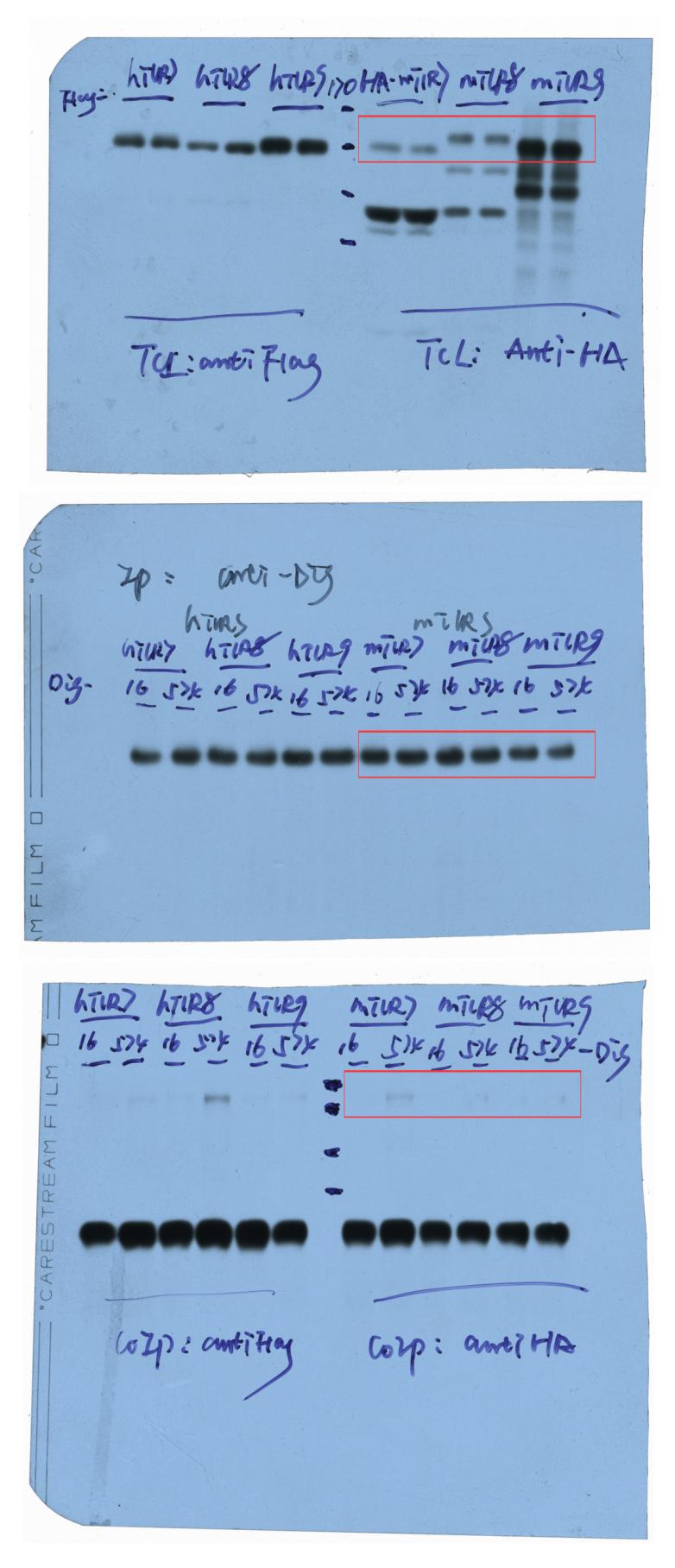


Full unedited gels for Figure 3***c***


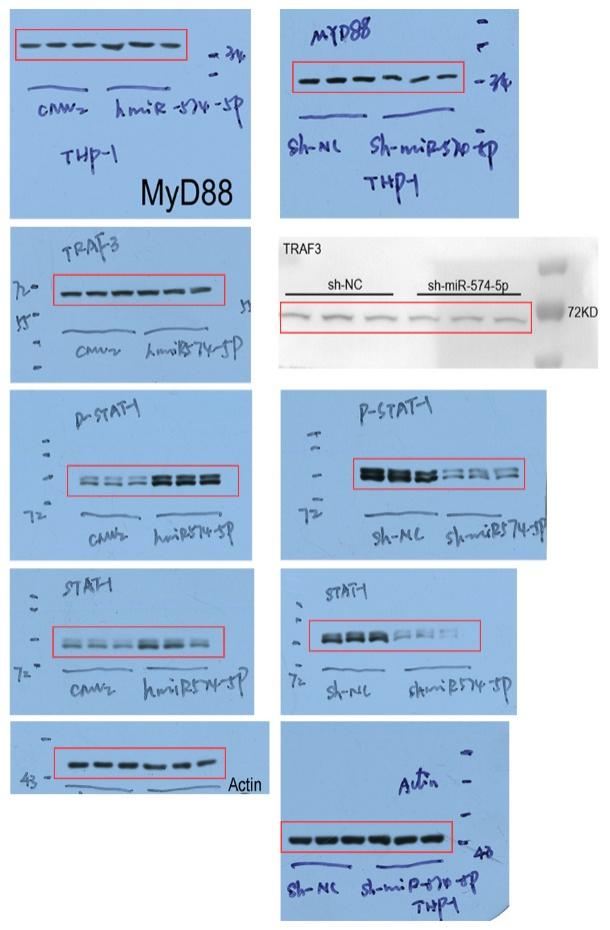


Full unedited gels for Figure 4***c***


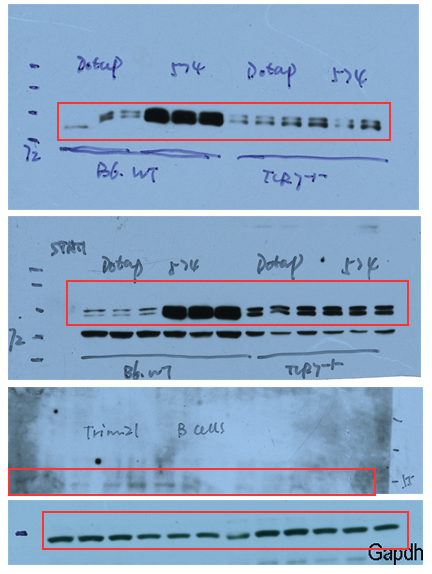


Full unedited gels for Figure 8***a***


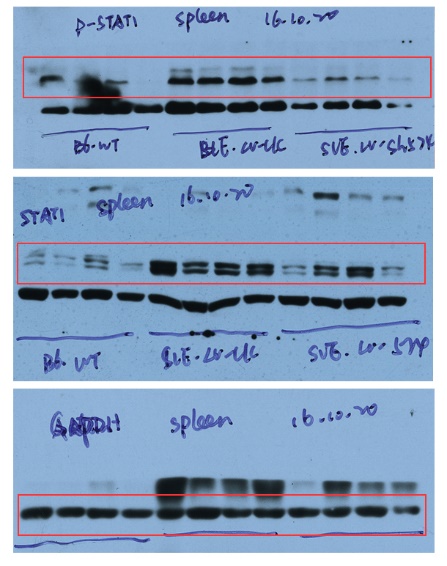


Full unedited gels for Figure S5***c***


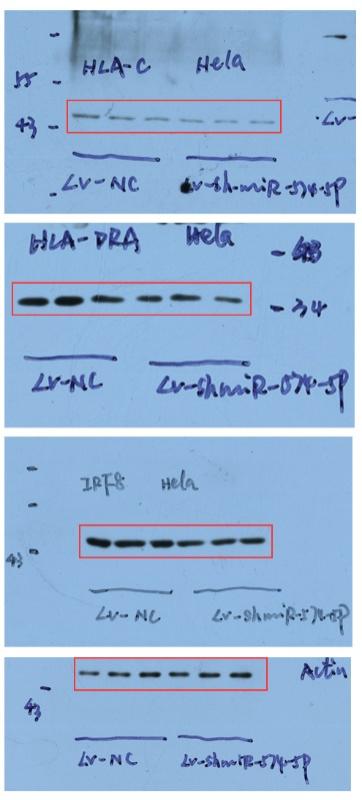

Supplement: Supplementary file 1 — Supplementary Material 1 [file 12964_2024_1601_MOESM1_ESM.docx]
